# Genetic polymorphism of IL-18 influences susceptibility to lung cancer in population of eastern China

**DOI:** 10.7150/jca.97039

**Published:** 2024-06-24

**Authors:** Xu Chen, Yanping Yao, Jianle Lao, Hua Li, Hailong Fu, Jun Qiu

**Affiliations:** 1 Center of Clinical Laboratory, First Affiliated Hospital of Soochow University, Suzhou, 215006, People's Republic of China.; 2 Department of Pharmacy, Suzhou Xiangcheng People's Hospital, Suzhou, Jiangsu, 215006, People's Republic of China.; 3 Department of cardiothoracic surgery, Affiliated Hospital of Youjiang Medical University for Nationalities, Baise, 533000, Guangxi Province, People's Republic of China.; 4 Key Laboratory of Tumor Molecular Pathology of Baise, Baise, 533000, Guangxi Province, People's Republic of China.

**Keywords:** IL-18, gene polymorphism, lung cancer, meta-analysis.

## Abstract

The association of Interleukin-18 (IL-18) genetic polymorphism with lung cancer risk has yielded inconsistent findings in previous studies. The current research aims to clarify the relationship of IL-18 gene polymorphism with lung cancer susceptibility through experimental investigation and meta-analysis, providing insights for lung cancer prevention and treatment. We conducted a thorough search of major databases from their inception until March 2024. OR and 95%CI were calculated to know the results of meta-analysis. The IL-18 gene polymorphism was detected using the PCR-RFLP method. Significant associations were detected across all genetic models in allele contrast (A vs. C: Odds Ratio [OR] = 1.29, 95% Confidence Interval [CI] = 1.07-1.55, p = 0.006), homozygote comparison (AA vs. CC: OR = 1.87, 95%CI = 1.34-2.62, p < 0.001), recessive genetic model (AA vs. CT/CC: OR = 1.54, 95%CI = 1.08-2.20, p = 0.018), and dominant genetic model (AA/AC vs. CC: OR = 1.41, 95%CI = 1.12-1.78, p = 0.003). Three genotypes (AA, AC, and CC) were identified for the IL-18 -607 C/A polymorphism, with significant associations noted for the AA genotype and A allele (p = 0.018 and 0.005, respectively). This is the first study which investigates this polymorphism with lung cancer in population of eastern China. The IL-18 -607 C/A polymorphism appears to significantly increase the risk of lung cancer in the population of Eastern China. Further research is imperative to validate these findings.

## Introduction

Lung cancer is the most devastating primary malignant tumor of the lungs, characterized by its high incidence and mortality rates. Based on the most recent statistics from 2020, lung cancer accounts for 11.4% of all new cancer cases, ranking second after breast cancer. Its mortality rate stands at 18%, the highest among all types of malignant tumors 1. These statistics also highlight significant gender disparities in lung cancer rates, with males experiencing a 14.3% incidence and a 21.5% mortality rate—both the highest among their gender. In contrast, females have an 8.4% incidence and a 13.7% mortality rate, placing them third and second, respectively, in terms of gender-specific cancer statistics 2, 3. The underlying causes of lung cancer remain elusive; however, current research suggests a complex interplay of factors. Beyond the well-documented contributions of smoking, chronic lung diseases, ionizing radiation, air pollution, and occupational hazards, there is a growing recognition of the critical roles played by familial clustering, genetic predisposition, immune system dysfunction, and disruptions in endocrine and metabolic processes for etiology of lung cancer.

Interleukin-18 (IL-18) is a multifaceted cytokine predominantly produced through the activation of mononuclear macrophages. It plays a critical role in inducing IFN-γ production and is intricately involved in the pathological mechanisms of various diseases, particularly in relation to cancer. IL-18 demonstrates a dualistic nature in cancer, exhibiting both tumor-suppressive and tumor-promoting effects.

On one hand, IL-18 promotes the production of IFN-γ and TNF-α, regulating the cytotoxic effects of NK cells in multiple ways and enhancing the Th-1 immune response. The produced IFN-γ and TNF-α have been shown to kill tumor cells *in vivo* 4-7. Notably, IL-18 significantly curbs tumor and tumor vascular growth in breast cancer cells and B16F10 melanocytes transfected with IL-18, an effect reliant on IFN-γ 8, 9. IFN-γ is known to induce the expression of anti-angiogenic chemotactic cytokines, including monokine and IP-10, and to suppress angiopoietin expression in tumor tissues, thereby inhibiting tumor blood vessel and tumor growth. During chemotherapy, the adverse effects of chemotherapeutic agents exacerbate cytokine imbalance in plasma. Supplementation with IL-18 in cancer patients has been shown to increase serum levels of cytokines like IL-2 and IFN-γ, enhancing NK cell cytotoxicity and overall immune cell function, which contributes to tumor growth and spread inhibition.

Conversely, emerging evidence suggests IL-18's role in promoting tumor development in certain contexts. Increased IL-18 levels have always been correlated with tumor clinicopathological features and prognosis. In patients with gastric cancer, significantly increased serum IL-18 levels serve as an important prognostic marker 10. Its expressions markedly elevated and play a significant role in tumor progression 11, 12. Cancer patients with distant metastases exhibit higher IL-18 expression compared with those without metastases and healthy controls 13. In gastric cancer cells, IL-18 has been shown to foster blood vessel formation, with vascular endothelial growth factor (VEGF) upregulating IL-18 expression through the extracellular signal-regulated kinase (ERK)1/2 pathway and reactive oxygen species (ROS) 14. Serum IL-18 levels in cancer patients correlate with VEGF levels, indicating a mutual induction mechanism where VEGF can stimulate IL-18 production, and IL-18, in turn, influences VEGF production via an autocrine pathway. Furthermore, tumor cells can escape immune surveillance through IL-18, as seen in lymphoid tissue where IL-18 induces mature activated NK cells to express the programmed death 1 gene (PD-1 gene), thereby evading immune detection.

The gene responsible for encoding IL-18 is located on chromosome 11 (11q22.2-q22.3). Too many SNPs of IL-18 gene have been discovered. Particularly, certain polymorphic sites within the gene's promoter region are linked to variations in gene transcription and protein expression levels. However, the -607 site is among the most widely investigated. A change from C to A at this site blocks the binding site for the cAMP response element-binding protein (CREB), leading to the dephosphorylation of CREB. This prevents CREB from binding to the cAMP response element (CRE) on DNA, consequently inhibiting gene transcription regulated by CRE. Research into the functional significance of this polymorphic site has indicated that it can influence the transcription and protein expression of IL-18 significantly.

Although research into the IL-18 -607 A/C polymorphism and its association with lung cancer susceptibility has been conducted in several countries and regions, the findings have been mixed and sometimes contradictory. Eastern China, known for its high lung cancer incidence rates, has yet to see reports on whether IL-18 gene polymorphism is linked to lung cancer susceptibility in this area. The -607 A/C polymorphism of the IL-18 gene is identified as a significant SNP locus by human genome mapping. Therefore, this study aims to investigate the relationship between the IL-18 gene's -607 A/C polymorphism and lung cancer susceptibility, employing both experimental approaches and meta-analysis. The findings are intended to serve as a basis for lung cancer prevention and treatment strategies.

## Materials and Methods

### Study subjects

Patients treated for lung cancer at our hospital from May 2018 to December 2023 were selected as the experimental group, with highly stringent inclusion criteria applied: (1) newly diagnosed cases of primary lung cancer, confirmed by an experienced pathologist; (2) patients who had not undergone any surgery, radiotherapy, chemotherapy, or molecular targeting therapy; (3) individuals with no history of other malignant tumors; (4) does not have any autoimmune disease, chronic diseases or severe metabolic disorders; (5) histopathological diagnosis of lung cancer. Inclusion criteria of control group: (1) no history of malignant tumor; (2) no history of autoimmune disease; (3) do not have any autoimmune diseases. Our laboratory staff used questionnaires and face-to-face interviews to investigate the subjects who met the criteria. The following information was collected: age, sex, alcohol consumption, smoking history, height and weight, with "alcohol consumption" defined as three or more times per week and over a period of 12 months. "Smoking" is defined as one or more cigarettes per day and smoking for more than 6 months. In addition, a body mass index (BMI) >=24kg/m 2 is used as a criterion for overweight and obesity. A total of 360 patients satisfied these criteria. Additionally, 630 healthy individuals were selected as the control group, all of whom had no history of tumors or infections and tested negative for alpha-fetoprotein and carcinoembryonic antigen. The Ethics Committee of First Affiliated Hospital of Soochow University has approved this study. All subjects, hailing from Jiangsu Province and without any kinship among them, as well as their families, have been informed about the study and have voluntarily signed informed consent forms.

### Gene polymorphism analysis

A single venous blood sample, approximately 3mL, was collected in the morning and stored in an EDTA anticoagulant tube for DNA extraction. The DNA was extracted using the Whole Blood DNA Purification Kit provided by Thermo Fisher Scientific, USA. Primer synthesis was carried out by Shanghai Tianhao Biotechnology Co., Ltd. The PCR-RFLP technique was employed for amplification, following protocols established in prior studies. Samples were analyzed using the ABI3730XL sequencer, and gene polymorphisms were examined with GeneMapper software version 4.1. The findings were further validated through sequencing.

### Meta-analysis process

For this meta-analysis, studies were rigorously searched for, reviewed, and data extracted by two seasoned authors. The search extended across prominent databases including the PubMed database, Cochrane Library, and Embase database. Critical information such as country, control source, sample size and Hardy-Weinberg Equilibrium (HWE) conformity were meticulously reviewed and extracted. The search keywords employed were “IL-18,” “single nucleotide polymorphism,” and “lung cancer.” The criteria for including and excluding studies were guided by methodologies outlined in previously published meta-analysis literature 15-23. Following strict adherence to the predetermined inclusion and exclusion criteria, the first and second authors carefully went through and assessed all available data and information. When the first and second writers couldn't agree on anything, they discussed it and came to a mutually agreed-upon decision by consulting the corresponding author. Three primary components comprised the main assessment criteria: the evaluation of exposure outcomes and variables (0-3 points); comparability between groups (0-2 points); and the selection of cases and controls (0-4 points). All methods utilized in this meta-analysis adhere to standards and protocols established in the literature 24-33.

### Statistical analysis

Statistical analyses for both the experimental component and the meta-analysis were conducted using SPSS 19.0 and STATA 11.0. The t-test and chi-square (χ²) test were employed to analyze measurement data and categorical data, respectively. A p-value of less than 0.05 was deemed statistically significant.

## Results

### General information of study subjects

Table 1 presents crucial information including gender, age, smoking status, alcohol consumption history, hypertension status, pathological types. The overview of this data suggests that there were no significant differences observed between the lung cancer group and the control group (P > 0.05).

### Genotyping and allele distribution of IL-18 gene -607 C/A polymorphism

For the IL-18 -607 C/A polymorphism, three genotypes (AA, AC, and CC) were identified. Significant associations were observed for the CC genotype and the C allele (all P < 0.05). Detailed information is provided in Table 2.

### Literature search

**Figure 1:**
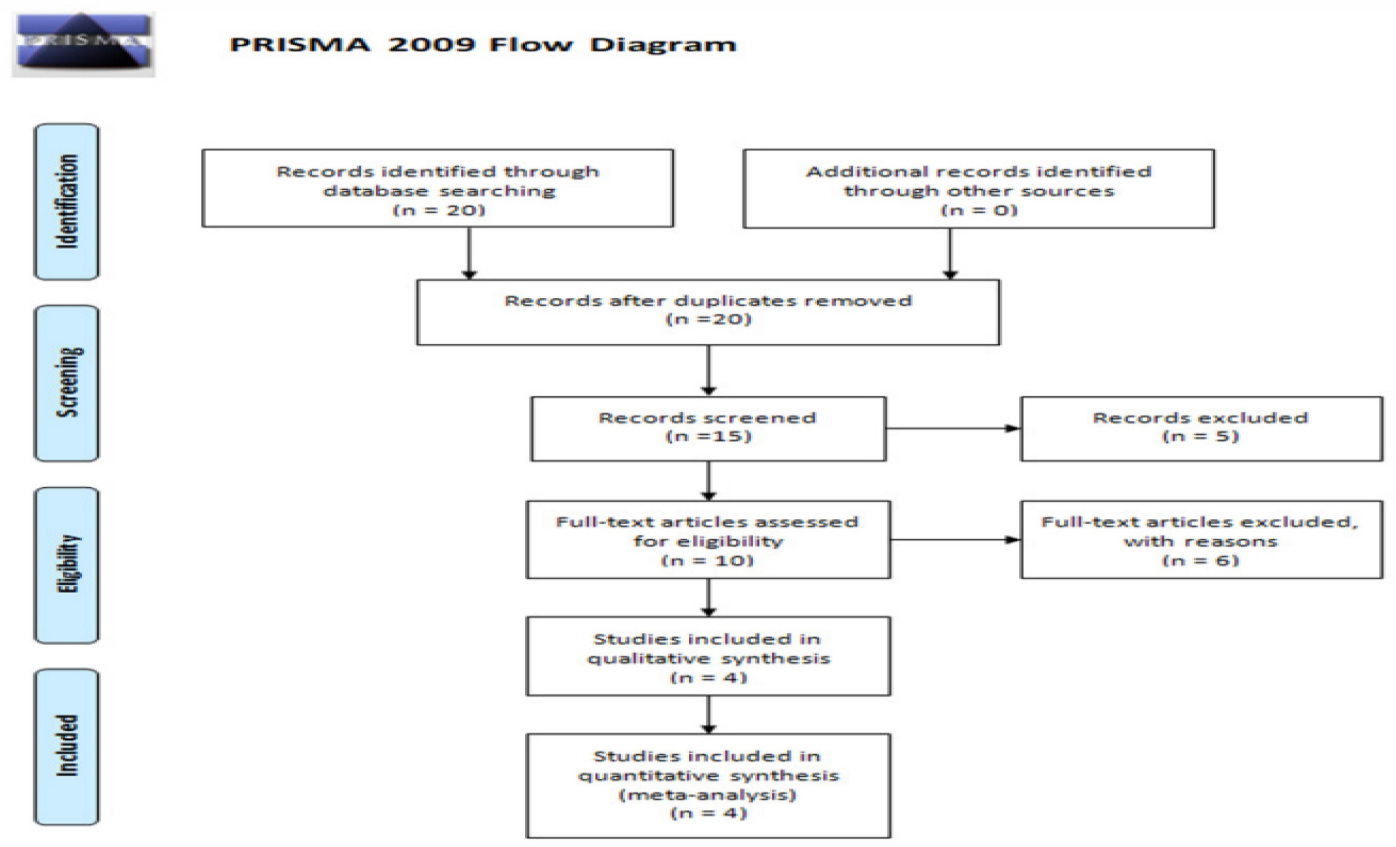
PRISMA 2009 Flow Diagram.

Figure 1 presents the flow diagram for the current meta-analysis, which included a total of four studies. Table 3 provides detailed information and data extracted from these studies 34-37. The populations studied in these four articles were Asian, showcasing varied sample sizes, genotyping methods, genotype frequencies, and allele frequencies. The quality assessment of the case-control studies, conducted using the Newcastle-Ottawa Scale, is displayed in Table 4.

### Allele and Genotype-wide Meta-analysis

**Figure 2:**
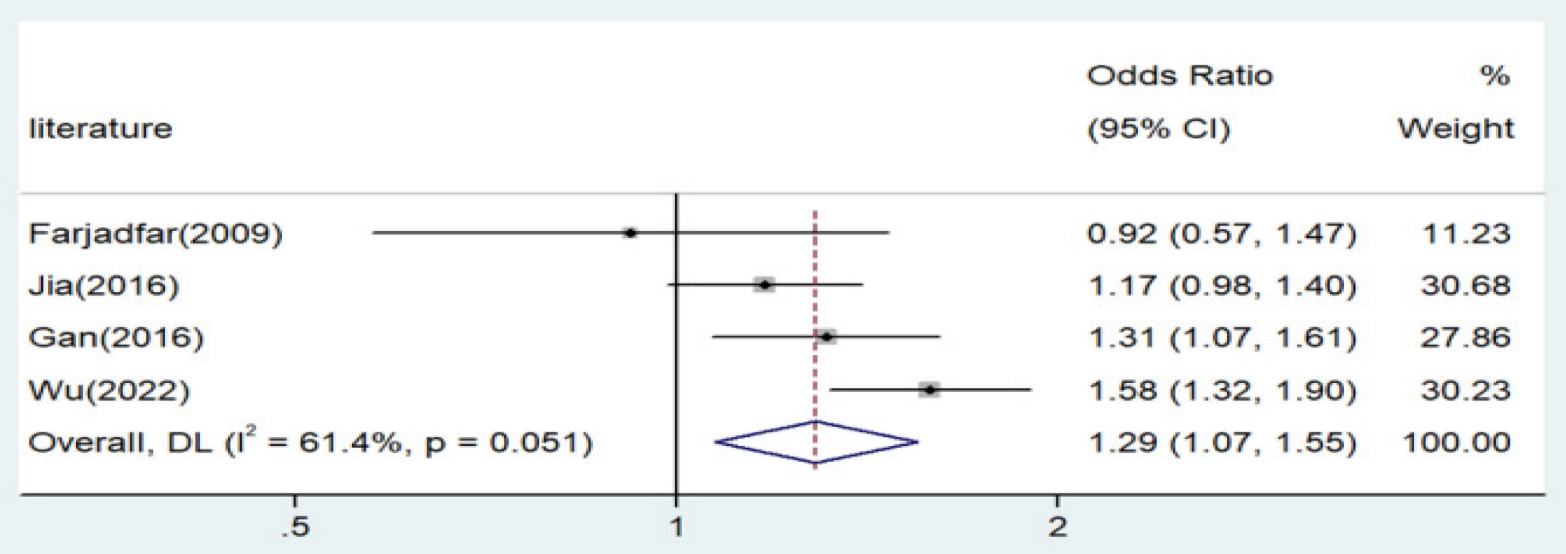
Forest plot for the associations between IL-18 gene -607 C/A polymorphism and lung cancer risk through allele contrast (A vs. C). OR, odds ratio; CI, confidence interval.

**Figure 3:**
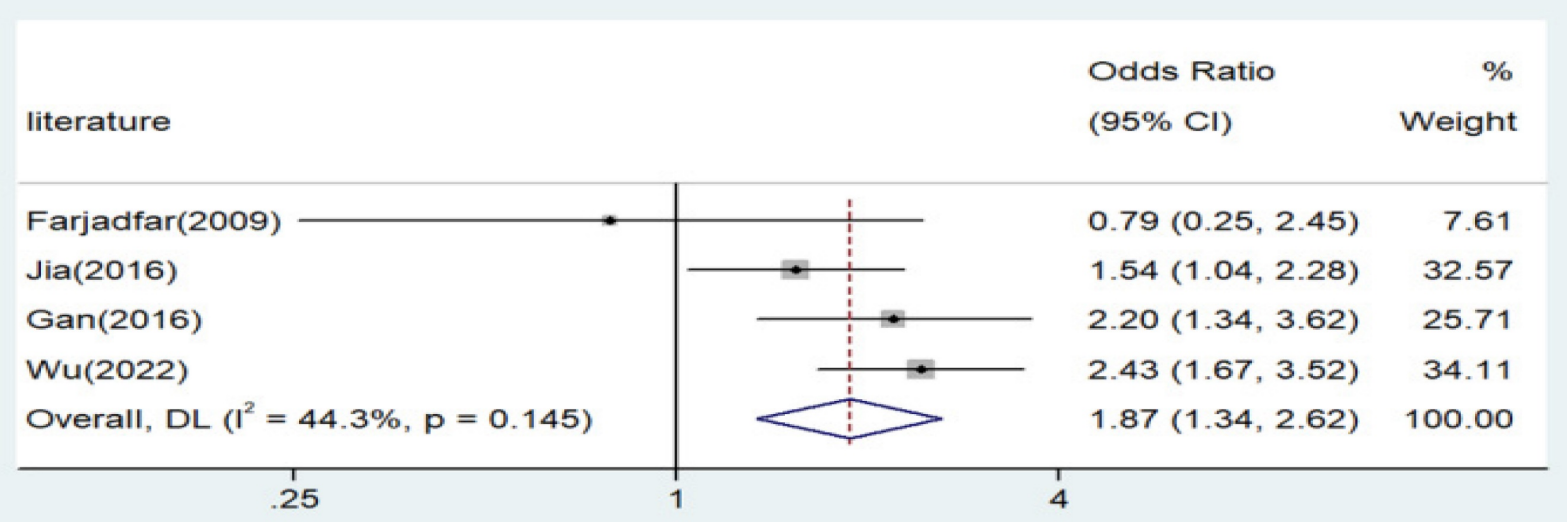
Forest plot for the associations between IL-18 gene -607 C/A polymorphism and lung cancer risk through homozygote comparison (AA vs. CC). OR, odds ratio; CI, confidence interval.

**Figure 4:**
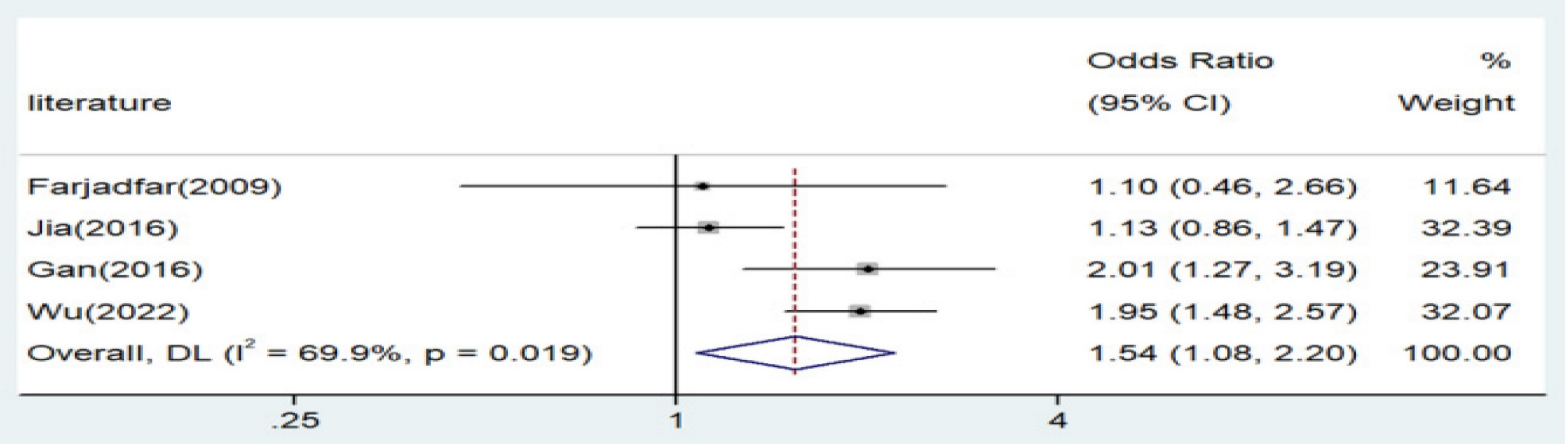
Forest plot for the associations between IL-18 gene -607 C/A polymorphism and lung cancer risk through recessive genetic model (AA vs. AC/CC). OR, odds ratio; CI, confidence interval.

**Figure 5:**
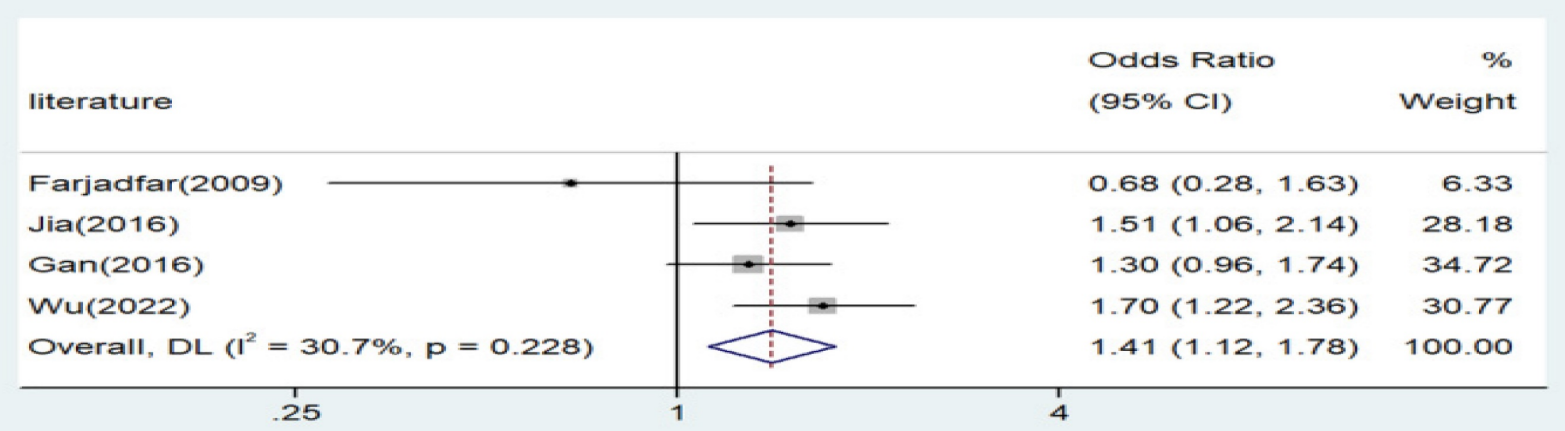
Forest plot for the associations between IL-18 gene -607 C/A polymorphism and lung cancer risk through dominate genetic model (AA/AC vs. CC). OR, odds ratio; CI, confidence interval.

Significant associations were observed across all genetic models, as depicted in Figures 2-5. The main findings relating the IL-18 -607 C/A polymorphism to lung cancer risk are summarized in Table 5. Our analysis revealed that the IL-18 gene -607 C/A polymorphism is associated with an increased risk of lung cancer. Furthermore, the CC genotype and the C allele were identified as risk factors for patients with lung cancer.

## Discussion

Epidemiological studies have identified several risk factors contributing to its rising incidence, including genetic predisposition, air pollution, occupational hazards, radiation exposure, dietary imbalances, and chronic inflammation. The swift advancements in molecular genetics have unearthed numerous genetic and epigenetic alterations intimately linked to lung cancer's onset and progression. It has been demonstrated that DNA genotypes can influence gene expression, with any alterations in DNA potentially leading to changes in amino acid sequences or proteins, thereby triggering disease. Single nucleotide polymorphisms (SNPs), characterized by single nucleotide changes in the genome, are the most prevalent form of mutation. Research indicates that if the frequency of a sequence variation exceeds 1% in a population, it is considered polymorphic. With deeper insights into molecular biology and the advancements in next-generation sequencing technologies, SNPs can now be precisely genotyped to unveil their associations with disease risk. Consequently, SNP sequencing is poised to play a pivotal role in diagnosing lung cancer and tailoring personalized treatment strategies.

The association between IL-18 gene polymorphism and lung cancer susceptibility has been reported by a large number of literatures, such as the literature published in 2019 38. But the site is different from ours. Our current investigation has revealed that the polymorphism is associated with an increased risk of lung cancer. As far as we know, this is the first study which investigates this polymorphism with lung cancer in population of eastern China. Our results are similar to those of previous studies 34, 37. They also IL-18 gene polymorphism contributes increased risk to the risk of lung cancer. Intriguingly, these findings contrast with those of two previously published studies that reported no association 35, 36. This discrepancy is not uncommon in genetic association research. Our study's location in Eastern China, which has a distinct temperature and climate compared to other parts of China, highlights the importance of geographical, regional, and environmental factors in genetic polymorphism. Moreover, genetic polymorphism is significantly influenced by population diversity. As China is home to 56 ethnic groups, the varied ethnic compositions across the northern, eastern, and southern regions of China naturally lead to differences in gene polymorphisms. This diversity underscores the complexity and variability of genetic influences on disease risk across different populations and regions.

We must acknowledge certain limitations in our current research. Firstly, we did not account for several confounding factors that could potentially influence our results. However, we plan to incorporate a broader range of epidemiological indicators in future studies to enhance the robustness of our research. Secondly, bioinformatics analysis has become a pivotal area in contemporary research 39-41, including the study of lung cancer. Moving forward, we aim to integrate gene polymorphism research with bioinformatic analysis. This approach will allow us to delve deeper into the pathogenesis of lung cancer, providing valuable insights for clinical diagnosis and prognosis.

In conclusion, the IL-18 -607 C/A polymorphism has been found to increase the risk of lung cancer in patients from Eastern China. There is an urgent need for future studies to validate our findings.

## Figures and Tables

**Table 1:** Participates characteristics of both lung cancer group and control group

Characteristics	Lung cancer group (N=360)	Control group (N=630)	p
Age	65.8±8.8	64.6±9.2	>0.05
Men	252(70.0%)	453(71.9%)	>0.05
BMI	25.8±2.0	24.9±1.5	>0.05
Smokers	78(21.7%)	129(20.5%)	>0.05
Drinking history	30(8.3%)	60(9.5%)	>0.05
Hypertension	48(13.3%)	75(11.9%)	>0.05
Pathological type			
Squamous cell Carcinoma	126(35.0%)		
Adenocarcinoma	144(40.0%)		
Small Cell Carcinoma	51(14.2%)		
Other	39(10.8%)		
Pathological stage			
I	19(5.3%)		
II	28(7.8%)		
III	98(27.2%)		
IV	215(59.7%)		

BMI, body mass index.

**Table 2:** Comparison of genotype and allele frequency between lung cancer group and control group

IL-18 -607 C/A	Control group (N=630)		Lung cancer group (N=360)		OR (95%CI)^ a^	P^a^
	n	Percentage (%)	n	Percentage (%)		
CC	198	31.4	72	20.0	1.00^REF^	
CA	192	30.5	108	30.0	1.55(0.83-2.88)	0.220
AA	240	38.1	180	50.0	2.06(1.16-3.66)	0.018
C	588	46.7	252	35.0	1.00^REF^	
A	672	53.3	468	65.0	1.63(1.17-2.25)	0.005

OR, odds ratio; CI, confidential index; ^a^Adjusted for sex and age by logistic regression model.

**Table 3:** Main characteristics of all case-control studies included in meta-analysis

Literatures	Ethnics (Country)	Genotyping methods	Source of control	Sample size	HWE conformity	Genotype frequency (Case)			Genotype frequency (Control)			Year
						CC	AC	AA	CC	AC	AA	
Farjadfar et al	Asian (Iran)	AS-PCR	PB	73/97	Yes	15	45	13	10	46	11	2009
Jia et al	Asian (China)	PCR- RFLP	PB	500/500	Yes	62	273	165	88	260	152	2016
Gan et al	Asian (China)	PCR-RFLP	PB	357/414	Yes	116	188	53	159	222	33	2016
Wu et al	Asian (Taiwan)	PCR-RFLP	PB	358/716	Yes	58	164	136	177	368	171	2022

PB: Population-based; HB: Hospital-based; HWE: Hardy-Weinberg equilibrium; RFLP: Restricted Fragment Length Polymorphism.

**Table 4:** Quality assessment of the case-control studies according to the Newcastle-Ottawa Scale

Literature	Selection of enrolled study subjects	Between-group comparability	Exposure outcomes and factors	Total
Farjadfar *et al.*	2	2	3	7
Jia *et al.*	3	3	3	9
Gan *et al.*	2	3	3	8
Wu *et al.*	3	3	3	9
Average	2.5	2.8	3.0	8.3

**Table 5:** Meta-analysis of the IL-18 -607 A/C polymorphism and lung cancer risk

Comparison	Population	N	Test of association			Mode	Test of heterogeneity		
			OR	95%CI	P		χ2	P	I^2^
A versus. C	Asian	4	1.29	1.07-1.55	0.006	Random	7.78	0.051	61.4
AA versus. CC	Asian	4	1.87	1.34-2.62	0	Fixed	5.39	0.145	44.3
AA versus. AC/CC	Asian	4	1.54	1.08-2.20	0.018	Random	9.97	0.019	69.9
AA/AC versus. CC	Asian	4	1.41	1.12-1.78	0.003	Random	4.33	0.228	30.7

OR, odds ratio; CI, confidence interval.
